# Quality indicators of clinical cancer care for prostate cancer: a population-based study in southern Switzerland

**DOI:** 10.1186/s12885-018-4604-2

**Published:** 2018-07-11

**Authors:** Laura Ortelli, Alessandra Spitale, Luca Mazzucchelli, Andrea Bordoni

**Affiliations:** 1 Ticino Cancer Registry, Cantonal Institute of Pathology, Via in Selva 24, 6600 Locarno, Switzerland; 2 Clinical Pathology, Cantonal Institute of Pathology, 6600 Locarno, Switzerland

**Keywords:** Quality of cancer care, Prostate cancer, Quality indicators, Cancer registry, Population-based study

## Abstract

**Background:**

Quality of cancer care (QoCC) has become an important item for providers, regulators and purchasers of care worldwide. Aim of this study is to present the results of some evidence-based quality indicators (QI) for prostate cancer (PC) at the population-based level and to compare the outcomes with data available in the literature.

**Methods:**

The study included all PC diagnosed on a three years period analysis (01.01.2011–31.12.2013) in the population of Canton Ticino (Southern Switzerland) extracted from the Ticino Cancer Registry database. 13 QI, approved through the validated Delphi methodology, were calculated using the “available case” approach: 2 for diagnosis, 4 for pathology, 6 for treatment and 1 for outcome. The selection of the computed QI was based on the availability of medical documentation. QI are presented as proportion (%) with the corresponding 95% confidence interval.

**Results:**

700 PC were detected during the three-year period 2011–2013: 78.3% of them were diagnosed through a prostatic biopsy and for 72.5% 8 or more biopsy cores were taken. 46.5% of the low risk PC patients underwent active surveillance, while 69.2% of high risk PC underwent a radical treatment (radical prostatectomy, radiotherapy or brachytherapy) and 73.5% of patients with metastatic PC were treated with hormonal therapy. The overall 30-day postoperative mortality was 0.5%.

**Conclusions:**

Results emerging from this study on the QoCC for PC in Canton Ticino are encouraging: the choice of treatment modalities seems to respect the international guidelines and our results are comparable to the scarce number of available international studies. Additional national and international standardisation of the QI and further QI population-based studies are needed in order to get a real picture of the PC diagnostic-therapeutic process progress through the definition of thresholds of minimal standard of care.

**Electronic supplementary material:**

The online version of this article (10.1186/s12885-018-4604-2) contains supplementary material, which is available to authorized users.

## Background

Prostate cancer (PC) is the most frequent cancer in men. In 2012, it represented almost 22% of all new cancer diagnoses in Europe and, despite the good prognosis, it is the second leading cause of death due to cancer [1]. In Switzerland, about 6200 PC cases are diagnosed annually, representing 30% of all tumours diagnoses. With a European age-standardized incidence rate of 158.6 cases per 100′000 inhabitants, the Switzerland is one of the countries with the highest incidence in Europe [1, 2]. The deaths pro year are about 1300, which corresponds to a European age-standardized mortality rate equal to 21.8 cases per 100′000 inhabitants [1, 2]. The 5-year survival rate is 88% and is comparable to other European countries [1].

Since the late ‘90s, in addition to survival analysis, studies about the Quality of Cancer Care (QoCC) became increasingly important to providers, regulators and purchasers of care worldwide as they strive to systematically measure and improve care [3]. QoCC studies performed using data of cancer registries evaluate and compare the quality of care at the population-based level giving a real description of the pattern of care at the regional level, without selection bias. Moreover, research on QoCC suggests that the progresses in the diagnostic and therapeutic methodology do not always reflect directly in the clinical practice and cases of underuse and overuse of care for cancer patients may occur [4, 5]. The data necessary for this kind of studies are available at the population-based Cancer Registries, so that, through specific quality indicators (QI), it is possible to document the delivered quality of care and provide regular feedback to healthcare workers and decision makers [6]. Moreover Cancer Registries give an independent description of the quality of care, without conflict of interest. All the structures involved in the oncological health care system in Ticino are strictly connected with the Ticino Cancer Registry allowing a complete coverage of the region.

The aim of the present study was to evaluate the results regarding 13 QI of the diagnostic and therapeutic process for PC diagnosed during the period 01.01.2011–31.12.2013 in Canton Ticino (Southern Switzerland) in order to assess the quality of PC care at the population-based level in comparison with the available literature. The 13 QI were produced by means of the Delphi process of a pool of QI derived through different guidelines. The oncological health care system in Canton Ticino includes five public hospitals, three private clinics with oncology and radiotherapy units and private oncological practices where PC patients usually undergo surgery and/or chemotherapy and/or radiotherapy. All the mentioned structures are connected with the Ticino Cancer Registry, allowing a direct access to the medical documentation necessary for the evaluation of QI and a complete coverage of the region in terms of data collection.

## Methods

### Data sources and case selection

All the resident population of Canton Ticino (346′539 inhabitants at 31.12.2013), the southern region of Switzerland, was included in the present study. Patients with a diagnosis of PC during the 3-year period 01.01.2011–31.12.2013 were considered eligible for the analysis. The cancer registry has a direct access to the inhabitants control office of canton Ticino, thus enabling the specific registration of patients resident in the region. For patients diagnosed or treated elsewhere we receive the corresponding documentation from other Swiss Cancer Registries. Cases were collected from the population-based Ticino Cancer Registry, which was founded in 1995 on the basis of a cantonal law and started the data collection in 1996. The Registry is part of the regional Institute of Pathology allowing the direct notification of the majority of new tumour cases; in addition, other cases are actively retrieved from public and private hospitals, radiology department, oncology centres, oncologists, general practitioners (urologists for the present study) and other Swiss Cancer Registries [7]. The information are prospectively retrieved, controlled and inserted in the Registry’s database by the Registry Staff on the basis of the guidelines of the International Agency for Research on Cancer (IARC) and the recommendations of the European Network of Cancer Registry (ENCR) [8, 9]. The Classification of Disease for Oncology (ICD-O-3) and the WHO Pathology and Genetics of Tumours of the Urinary System and Male Genital Organs are used to classify the site and the histological type of the tumour, whereas the stage is registered according to the 7th edition of the American Joint Committee on cancer (AJCC) Staging Manual [10, 11]. In order to check the validity and consistency of data, quality controls are periodically performed through several internal tests, the IARC check program (IARCcrgTools) and the Joint Research Centre – ENCR quality check software [12–14]. Standard indicators and the method of Bullard et al. are used to assess the case completeness [7, 15, 16]. Each QI was the result of an accurate data collection, performed by specific-trained data-managers, who extracted the necessary information directly from the medical documentation, thus avoiding misleading interpretations and permitting a uniform codification, necessary to achieve a good comparability level.

The D’Amico risk classification was used to define and stratify localized PC (N0, M0) for the analysis of treatment modalities (i.e. QI7–9), as following:

- low risk PC: PSA ≤10 ng/ml and Gleason score ≤ 6 and clinical stage cT1-cT2a;
- intermediate risk PC: 10 ng/ml < PSA < 20 ng/ml or Gleason score = 7 or clinical stage cT2b-cT2c;
- high risk PC: PSA ≥ 20 ng/ml or Gleason score ≥ 8 or clinical stage cT3-cT4.

### List of quality indicators and analysis

This study is part of a larger prospective, descriptive, population-based study on the QoCC in Canton Ticino, southern Switzerland, named QC_3_ project, with the aim to identify and compute QI for the following tumour localizations: colon-rectum, prostate, lung and ovary/endometrium. The methodology used to select QI is in depth described in Bianchi et al. [17]. Here, a brief summary of the entire procedure. For each tumour site, we initially selected a preliminary list of evidence-based QI, through a comprehensive literature research, taking into account their degree of relevance and feasibility. A multidisciplinary team enclosing local specialists of the main medical disciplines (radio-oncology, urology for PC, oncology and pathology) selected and approved QI through the validated Delphi methodology. QI were then submitted to an independent external international multidisciplinary Advisory Board. QI achieving an agreement greater than 70% were finally approved. Only those QI whose collection was evaluated to be “feasible at the population-based level” were retained. The definitive list for PC contains 23 QI, distributed in the following clinical domains: 6 for diagnosis and staging, 4 for pathology, 9 for treatment and 4 for outcome and follow-up (Additional file 1). For the present study, we computed a core of 13 QI for which all the medical documentation needed for data collection was already available at the Ticino Cancer Registry or a minor data collection was still needed to complete the analysis: 2 for diagnosis, 4 for pathology, 6 for treatment modalities and 1 for outcome (Table 1).

**Table 1:** Quality indicators (QI) for patients diagnosed with prostate cancer (PC) in canton Ticino, Southern Switzerland, between 01.01.2011 and 31.12.2013

QUALITY INDICATOR		NUMERATOR		DENOMINATOR		%^b^ CI (95%)	MEDICAL DOCUMENTATION	RATIONALE
		Description	N	Description	N			
QI1	Proportion of patients with prostate cancer and the diagnosis based on prostatic biopsy^a^.	Number of patients with prostate cancer whose diagnosis was based on prostate biopsy^a^.	535	Number of patients with prostate cancer.	700 of which 683 with available information.	78.3% (75.2%;81.4%)	Biopsy pathology report. Reports/discharge letters coming from all hospitals units/departments (i.e. surgery, medicine, radiation oncology, medical oncology).	Biopsy is the recognised diagnostic procedure.
		*(17 missing: information not retrieved)*						
QI2	Proportion of patients with prostate cancer and 8 or more diagnostic prostatic biopsies.	Number of patients with prostate cancer who underwent 8 or more diagnostic prostatic biopsies.	377	Number of patients with prostate cancer whose diagnosis was based on prostatic biopsy.	535 of which 520 with available information.	72.5% (68.7%;76.3%)	Biopsy pathology report. Request form of pathology examination.	A sufficient number of prostatic biopsies is required for an adequate sampling of the tissue.
		*(15 missing: information not retrieved)*						
QI3	Proportion of patients with prostate cancer and the pathology report of the biopsy including the following characteristics: - histologic type according to WHO - histologic grade with Gleason score - tumour quantitation (number of positive cores/total number of cores and/or proportion of prostatic tissue involved by tumour).	Number of patients with prostate cancer whose pathology report of the biopsy included the histologic type according to WHO.	498	Number of patients with prostate cancer undergoing biopsy.	535 of which 528 with available information.	94.3% (92.3%;96.3%)	Biopsy pathology report.	Standardization of microscopic tumour characteristics is necessary for an adequate treatment planning.
		*(7 missing: information not retrieved)*						
		Number of patients with prostate cancer whose pathology report of the biopsy included the histologic grade with Gleason score.	533	Number of patients with prostate cancer undergoing biopsy.	535 of which 534 with available information.	99.8% (99.5%;100.0%)		
		*(1 missing: information not retrieved)*						
		Number of patients with prostate cancer whose pathology report of the biopsy included tumour quantitation (number of positive cores/total number of cores and/or proportion of prostatic tissue involved by tumour).	524	Number of patients with prostate cancer undergoing biopsy.	535 of which 530 with available information.	98.9% (98.0%;99.8%)		
		*(5 missing: information not retrieved)*						
QI4	Proportion of patients with prostate cancer and the pathology report of the TUR-P including the following characteristics: - histologic type according to WHO - histologic grade with Gleason score - tumour quantitation (proportion of prostatic tissue involved by tumour).	Number of patients with prostate cancer whose pathology report of the TUR-P included the histologic type according to WHO.	74	Number of patients with prostate cancer undergoing TUR-P.	88 of which 81 with available information.	91.4% (85.2%;97.5%)	TUR-P pathology report.	Standardization of microscopic tumour characteristics is necessary for an adequate treatment planning.
		*(7 missing: information not retrieved)*						
		Number of patients with prostate cancer whose pathology report of the TUR-P included the histologic grade with Gleason score.	79	Number of patients with prostate cancer undergoing TUR-P.	88 of which 84 with available information.	94.1% (89.0%;99.1%)		
		*(4 missing: information not retrieved)*						
		Number of patients with prostate cancer whose pathology report of the TUR-P included the tumour quantitation (number of positive cores/total number of cores and/or proportion of prostatic tissue involved by tumour).	59	Number of patients with prostate cancer undergoing TUR-P.	88 of which 81 with available information.	72.8% (63.1%;82.5%)		
		*(7 missing: information not retrieved)*						
QI5	Proportion of patients with prostate cancer whose pathology report of the prostatectomy with pelvic lymphadenectomy includes the number of resected lymph nodes.	Number of patients with prostate cancer whose pathology report of the prostatectomy with pelvic lymphadenectomy included the number of resected lymph nodes.	195	Number of patients with prostate cancer undergoing prostatectomy with pelvic lymphadenectomy.	197 of which 195 with available information.	100.0% (100%;100%)	Prostatectomy pathology report.	Standardization of microscopic tumour characteristics is necessary for an adequate treatment planning.
		*(2 missing: information not retrieved)*						
QI6	Proportion of patients with prostate cancer and the pathology report of the prostatectomy including the following characteristics: - histologic type according to WHO - histologic grade with Gleason score - extraprostatic extension - seminal vescicle invasion - margins status - pathologic staging (pTNM) according to AJCC TNM 7th ed.	Number of patients with prostate cancer whose pathology report of the prostatectomy included the histologic type according to WHO.	198	Number of patients with prostate cancer undergoing prostatectomy.	220 of which 215 with available information.	92.1% (88.5%;95.7%)	Prostatectomy pathology report.	Standardization of microscopic tumour characteristics is necessary for an adequate treatment planning.
		*(5 missing: information not retrieved)*						
		Number of patients with prostate cancer whose pathology report of the prostatectomy included the histologic grade with Gleason score.	219	Number of patients with prostate cancer undergoing prostatectomy.	220 of which 219 with available information.	100.0% (100%;100%)		
		*(1 missing: information not retrieved)*						
		Number of patients with prostate cancer whose pathology report of the prostatectomy included the extraprostatic extension.	198	Number of patients with prostate cancer undergoing prostatectomy.	220 of which 215 with available information.	92.1% (88.5%;95.7%)		
		*(5 missing: information not retrieved)*						
		Number of patient with prostate cancer whose pathology report of the prostatectomy included the seminal vescicle invasion.	210	Number of patients with prostate cancer undergoing prostatectomy.	220 of which 215 with available information.	97.7% (95.7%;99.7%)		
		*(5 missing: information not retrieved)*						
		Number of patients with prostate cancer whose pathology report of the prostatectomy included the margins status.	216	Number of patients with prostate cancer undergoing prostatectomy.	220 of which 220 with available information.	98.2% (96.4%;100.0%)		
		*(0 missing: information not retrieved)*						
		Number of patients with prostate cancer whose pathology report of the prostatectomy included the pathologic staging (pTNM) according to AJCC TNM 7th ed.	219	Number of patients with prostate cancer undergoing prostatectomy.	220 of which 220 with available information.	99.6% (98.7%;100.0%)		
		*(0 missing: information not retrieved)*						
QI7	Proportion of patients with localized (N0, M0) low risk (cT1–2a and Gleason≤6 and PSA ≤ 10 ng/ml) prostate cancer undergoing active surveillance.	Number of patients with localized (N0, M0) low risk (cT1–2a and Gleason≤6 and PSA ≤ 10 ng/ml) prostate cancer who underwent an active surveillance.	33	Number of patients with localized (N0, M0) low risk (cT1–2a and Gleason≤6 and PSA ≤ 10 ng/ml) prostate cancer.	84 of which 71 with available information.	46.5% (34.9%;58.1%)	Reports/discharge letters coming from all hospitals units/departments (i.e. surgery, medicine, radiation oncology, medical oncology).	Guidelines indicate active surveillance as preferred treatment for low risk prostate cancers.
		*(13 missing: information not retrieved)*						
QI8	Proportion of patients with localized (N0, M0) high risk (cT3–4 or Gleason≥8 or PSA ≥ 20 ng/ml) prostate cancer undergoing radical treatment (radical prostatectomy ± pelvic lymphadenectomy, RT or brachytherapy).	Number of patients with localized (N0, M0) high risk (cT3–4 or Gleason≥8 or PSA ≥ 20 ng/ml) prostate cancer who underwent a radical treatment (radical prostatectomy ± pelvic lymphadenectomy, RT or brachytherapy).	144	Number of patients with localized (N0, M0) high risk (cT3–4 or Gleason≥8 or PSA ≥ 20 ng/ml) prostate cancer.	248 of which 208 with available information.	69.2% (63.0%;75.5%)	Reports/discharge letters coming from all hospitals units/departments (i.e. surgery, medicine, radiation oncology, medical oncology).	Guidelines indicate prostatectomy as 1st line treatment for high risk prostate cancers.
		*(40 missing: information not retrieved)*						
QI9	Proportion of patients with localized (N0, M0) high risk (cT3–4 or Gleason≥8 or PSA ≥ 20 ng/ml) prostate cancer undergoing radical RT with neo-adjuvant HT.	Number of patients with localized (N0, M0) high risk (cT3–4 or Gleason≥8 or PSA ≥ 20 ng/ml) prostate cancer who underwent a neo-adjuvant HT before radical RT.	66	Number of patients with localized (N0, M0) high risk (cT3–4 or Gleason≥8 or PSA ≥ 20 ng/ml) prostate cancer undergoing radical RT.	75 of which 75 with available information.	88.0% (80.7%;95.4%)	Reports/discharge letters coming from all hospitals units/departments (i.e. surgery, medicine, radiation oncology, medical oncology).	In high risk prostate cancer, treated with RT, the use of neo-adjuvant HT improves overall survival.
		*(0 missing: information not retrieved)*						
QI10	Proportion of patients with non-metastatic (M0) pT2 or pT3 prostate cancer undergoing prostatectomy ± pelvic lymphadenectomy with uninvolved margins^a^.	Number of patients with non-metastatic (M0) pT2 or pT3 prostate cancer who underwent prostatectomy ± pelvic lymphadenectomy with uninvolved margins.	97 (pT2) 40 (pT3)	Number of patients with non-metastatic (M0) pT2 or pT3 prostate cancer undergoing prostatectomy ± pelvic lymphadenectomy.	118 (pT2)/97 (pT3) of which 118/97 with available information.	82.2% (pT2) (75.3%;89.1%) 41.2% (pT3) (31.4%;51.0%)	Prostatectomy pathology report.	Margins status is the only criteria for evaluating the radicality of the prostatectomy.
		*(0 missing: information not retrieved)*						
QI11	Proportion of patients with non-metastatic (M0) prostate cancer undergoing external beam RT with dose escalation to at least 74 Gy.	Number of patients with non-metastatic (M0) prostate cancer who underwent an external beam RT with dose escalation to at least 74 Gy.	148	Number of patients with non-metastatic (M0) prostate cancer undergoing RT.	198 of which 194 with available information.	76.3% (70.3%;82.3%)	Reports/discharge letters coming from all hospitals units/departments (i.e. surgery, medicine, radiation oncology, medical oncology).	RT dose escalation between 74 and 78 Gy increases biochemical control of prostate cancer.
		*(4 missing: information not retrieved)*						
QI12	Proportion of patients with metastatic (M1) prostate cancer undergoing immediate (within 3 months from the diagnosis) HT or bilateral orchiectomy.	Number of patients with metastatic (M1) prostate cancer who underwent HT or bilateral orchiectomy within 3 months from the diagnosis.	50	Patients with metastatic (M1) prostate cancer.	74 of which 68 with available information.	73.5% (63.0%;84.0%)	Reports/discharge letters coming from all hospitals units/departments (i.e. surgery, medicine, radiation oncology, medical oncology).	HT is the indicated treatment for metastatic prostate cancer.
		*(6 missing: information not retrieved)*						
QI13	Proportion of patients with non-metastatic (M0) prostate cancer dead within 30 days after prostatectomy ± pelvic lymphadenectomy (post-operative mortality).	Number of patients with non-metastatic (M0) prostate cancer who died within 30 days after prostatectomy ± pelvic lymphadenectomy.	1	Number of patients with non-metastatic (M0) prostate cancer undergoing prostatectomy ± pelvic lymphadenectomy.	219 of which 219 with available information.	0.5% (0.0%; 1.4%)	Reports/discharge letters coming from all hospitals units/departments (i.e. surgery, medicine, radiation oncology, medical oncology).	Indicator for the outcome after radical prostatectomy.
		*(0 missing: information not retrieved)*						

Abbreviation: *PSA* prostate specific antigen, *TUR-P* transurethral prostatic resection, *AJCC* America Joint Committee on Cancer; *WHO* World Health Organization, *HT* hormonal therapy, *RT* radiotherapy, *ChT* chemotherapy

^a^The TUR-P was not considered as biopsy

^b^The proportion was calculated on the basis of the available/retrieved information (i.e. missing cases were excluded)

Each QI was defined through a numerator, i.e. the number of patients who fulfilled the specific criteria, and a denominator, i.e. the number of eligible patients. The proportion (%) and the relative 95% confidence interval (95% CI) were calculated based on the binomial distribution. Cases identified only by a death certificate (i.e. DCO cases, 0.99%) were excluded from the present analysis. The “available case analysis” approach was used, i.e. cases for which we could not retrieve the information in the consulted medical documentation were excluded from the numerator as well from the denominator of the QI and classified as “missing”.

For QIs concerning prostatic biopsies (i.e. QI1, QI2 and QI3), the transurethral prostatic resections (TUR-P) were not included in the analysis. Moreover, for QIs concerning patients operated on (QI5, QI6, QI8, QI10 and QI13), only surgical interventions performed within 6 months from the diagnosis were considered for the calculation.

For comparative goals, publications on QI were identified and selected by means of a literature search in PubMed/MEDLINE, using initially general or specific keywords/expressions and a combination of them, such as the followings: “population-based study”, “quality indicators”, “quality of care or quality of cancer care”, “prostate cancer”, “low risk or high risk or intermediate risk”, “diagnosis”, “biopsy”, “pathology report”, “TUR-P”, “prostatectomy”, “treatment”, “active surveillance”, “radiotherapy”, “dose escalation radiotherapy”, “radical”, “neo-adjuvant or neoadjuvant or preoperative treatment”, “hormone therapy”, “metastatic”, “surgical margins”, “outcomes”, “survival”, “postoperative mortality”, “30-day mortality”. All the peer-reviewed articles were included, except case reports, letters, abstracts or editorials.

SAS system version V9.3 (SAS Institute Inc., Cary, North Carolina, USA) was used for the analysis.

## Results

Between 01.01.2011 and 31.12.2013, the Ticino Cancer Registry registered 700 diagnoses of PC. The median age at the diagnosis was 70.5 years (range: 36–100). The results for the 13 selected QI are reported in Table 1 with the following information: QI definition, numerator and denominator description (selection criteria and corresponding numbers), QI results, i.e. percentage (%) with the corresponding 95% CI, list of the medical documentation analysed to retrieve the necessary information and the QI rationale.

The number of “missing”, i.e. cases for whom we were not able to retrieve the needed information, was low (under 10%) for almost all QI, with the exceptions of QI7 and QI8, for which we could not retrieve the performed treatment modality in the available medical reports for 15.5% (*N* = 13) and 16.1% (*N* = 40) of patients, respectively.

### Quality indicators for diagnosis

QI1–2 refer to the clinical domain of the diagnosis. Overall, 535 PC were confirmed through a needle biopsy (78.3%; 95%CI: 75.2%; 81.4%) (QI1) and in 377 cases (72.5%; 95%CI: 68.7%; 76.3%) 8 or more biopsy cores were taken (QI2).

### Quality indicators for pathology

QI3–6 refer to the pathology clinical domain. In our study, the biopsy pathological reports described the histology of the tumour according to the WHO definition in 498 PC (94.3%; CI95%: 92.4%; 96.3%), the differentiation grade according to Gleason score in 533 PC (99.8%; CI95%: 99.5%; 100.0%) and the proportion of sample’s tissue involved by the tumour or the number of positive cores on the total number of taken specimens in 524 PC (98.9%; CI95%: 98.0%; 99.8%) (QI3). As expected by the guidelines of the College of American Pathologists and the European Society of Uropathology, we found out the following information in the pathological reports of the transurethral prostatic resection (TUR-P) (QI4): the histology description according to the WHO classification was reported in 91.4% of cases (CI95%: 85.2%; 97.5%), the histologic grade according to the Gleason score in 94.1% (CI95%: 89.0%; 99.1%) and the proportion of tissue involved by the tumour in 72.8% (CI95%: 63.1%; 82.5%) [18, 19]. The number of resected lymph nodes was reported in all patients undergoing prostatectomy with pelvic lymphadenectomy (QI5). Among the 220 patients undergoing prostatectomy with or without pelvic lymphadenectomy, the pathology report (QI6) included the information on the histological type according to WHO for 198 cases (92.1%; CI95%: 88.5%; 95.7%), the histological grade according to the Gleason score for all cases, the extraprostatic extension for 198 (92.1%; CI95%: 88.5%; 95.7%), the presence of seminal vescicles invasion for 210 (97.7%; CI95%: 95.7%; 99.7%), the margins status for 216 (98.2%; CI95%: 96.4%; 100.0%) and the pathological stage of the disease according to the AJCC TNM 7th edition for 219 (99.6%; CI95%: 98.7%; 100.0%) [11].

### Quality indicators for treatment

QI7–12 refer to treatment modalities for PC and assess the compliance with the European guidelines [20, 21]. QI7–9 stratify localized prostate cancers (N0, M0) according to the D’Amico classification of risk and recurrence. Thirty-three patients (46.5%; CI95%: 34.9%; 58.1%) with a low risk PC underwent active surveillance (QI7) and 144 patients (69.2%; CI95%: 63.0%; 75.5%) with a high risk PC were treated radically through prostatectomy (with or without pelvic lymphadenectomy), radiotherapy or brachytherapy (QI8). Furthermore, 66 patients (88.0%; CI95%: 80.7%; 95.4%) with a high risk PC undergoing radiotherapy performed in addition a neo-adjuvant hormonal treatment (QI9). Among PC patients undergoing radical prostatectomy (with or without pelvic lymphadenectomy), 97 with stage pT2 (82.2%; CI95%: 75.3%; 89.1%) and 40 with pT3 (41.2%; CI95%: 31.4%; 51.0%) had uninvolved margins (QI10). 148 (76.3%; CI95%: 70.3%; 82.3%) patients with localized non-metastatic PC (M0) treated with external beam radiotherapy underwent a dose escalation to at least 74 Gy (QI11). QI12 analysed the treatment for metastatic (every T, every N, M1) PC: 50 patients (73.5%; CI95%: 63.0%; 84.0%) underwent hormonal therapy within 3 months from the date of the diagnosis.

### Quality indicator for outcome

QI13 evaluated the outcome for non-metastatic (M0) PC after the radical prostatectomy (with or without pelvic lymphadenectomy), accounting only 1 death within 30 days from the surgical intervention (0.5%; CI95%: 0.0%; 1.4%).

## Discussion

The present population-based study allowed to evaluate the QoCC for PC and to find out possible weaknesses in the care system of canton Ticino, southern Switzerland. PC diagnosis and treatment reflect the recommendations of the guidelines and state a good quality level of the cancer care in our region compared with other countries. Particularly, the 69% of high-risk PC patients underwent radical treatment (72% in the U.S. and 66% in Australia) whereas 88% of them benefited of neo-adjuvant HT in addition to RT (92% in Sweden) [22–24]. On the other hand, there is still room for improvement regarding HT for metastatic patients (73% in southern Switzerland versus 88% in Sweden) and for prostatectomy specimens’ margins, which were uninvolved in 82% pT2 cases in our region (92% in Germany) [24, 25].

A first strength of this study is the selection procedure of the QI, described in Bianchi et al. [17]: the validated Delphi methodology together with local and international experts’ advices assured an adequate evaluation of relevance, validity and feasibility of QI. In addition, a possible selection bias is avoided and a representation of the entire regional health care system is guaranteed, thanks to the population-based data collection and the Cancer Registry access to public and private data sources. According to Lorez et al. the registration completeness for PC in canton Ticino is 87.3% (mean value for all Swiss cancer registries is 87.9%), confirming a satisfactory coverage level [26]. The direct extraction of information from the original medical documentation assures a homogeneous codification as well as a high grade of coherence.

A limit of the present study could be the lack of information for a few QI. Particularly for QI7 and QI8 we observed a large percentage of “missing” cases. In general, we had some difficulties in getting the complete medical documentation needed to assess the performed therapeutic treatment prospectively. One reason could be that some patients underwent PC treatments outside of canton Ticino (i.e. in other cantons), hence there was a consistent time gap before getting the needed information. Consequently, for these QI we had larger 95% CI, possibly affecting the statistical power of the analysis. This could be solved extending the observation period or the population at risk for future projects and could be a strong motivation to stimulate and improve the communication among public and private facilities and cancer registries. Furthermore, we faced some difficulties in finding comparison data in the literature, particularly at the population-based level, because of different selection criteria, surgical procedures and pathological protocols, heterogeneous adhesion to specific national/international guidelines as well as general lack in QI definition and standardization; these aspects could limit the potential value of such studies. Another limitation of the study could be the literature research performed to select comparative studies that could have missed some relevant studies.

In the following paragraph we describe the results of each QI in comparison with similar data in the available literature.

**Fig. 1:**
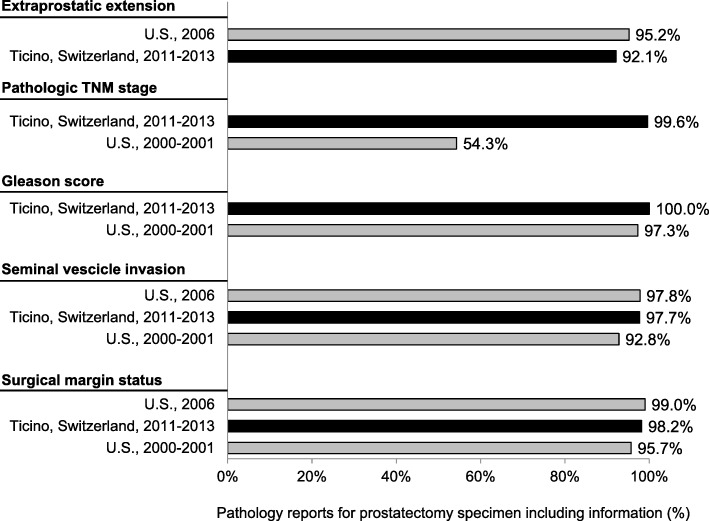
QI6. Completeness of pathology reports for prostatectomy specimens: comparison between southern-Switzerland and U.S

**Fig. 2:**
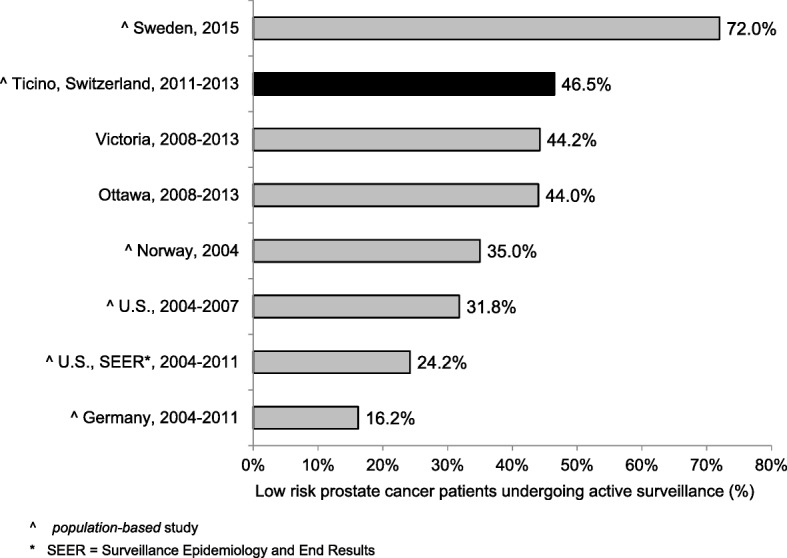
QI7. Proportion of patients with low-risk prostate-cancer undergoing active-surveillance: comparison between southern-Switzerland and other countries

**Fig. 3:**
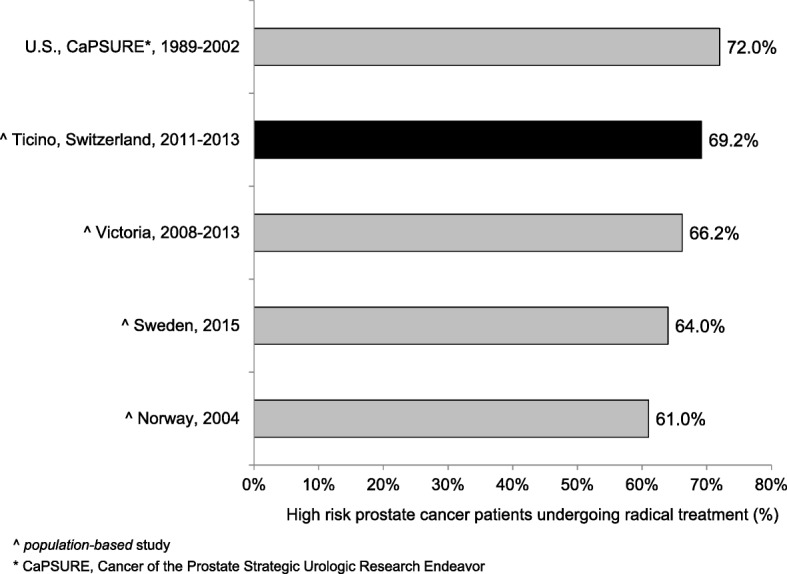
QI 8. Proportion of patients with high-risk prostate-cancer undergoing radical treatment: comparisons between southern-Switzerland and other countries

**Fig. 4:**
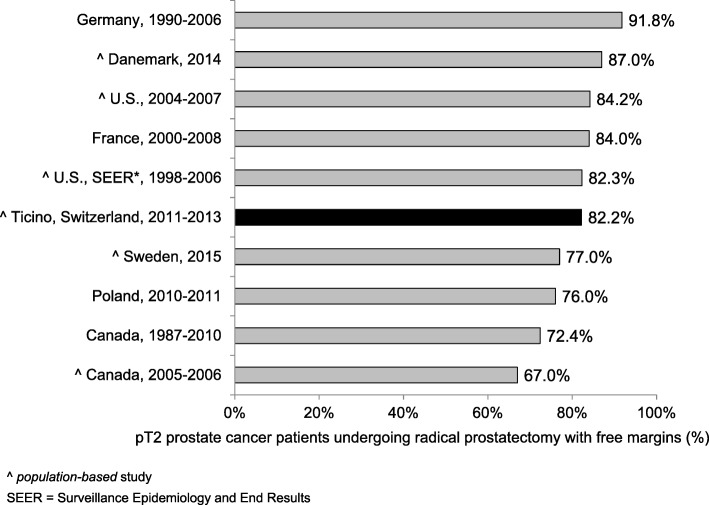
QI10. Proportion of patients with pT2 prostate-cancer with free-margins after radical-prostatectomy: comparison between southern-Switzerland and other countries

**Fig. 5:**
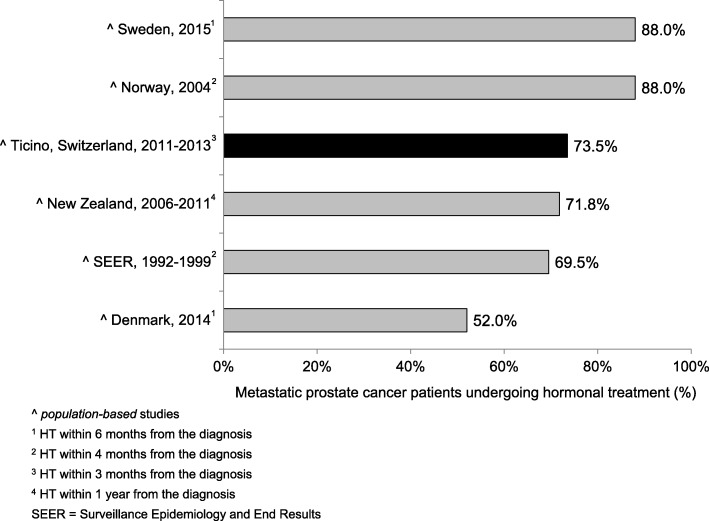
QI12. Proportion of patients with metastatic prostate-cancer (M1) treated with hormonal-therapy: comparison between southern-Switzerland and other countries

**Fig. 6:**
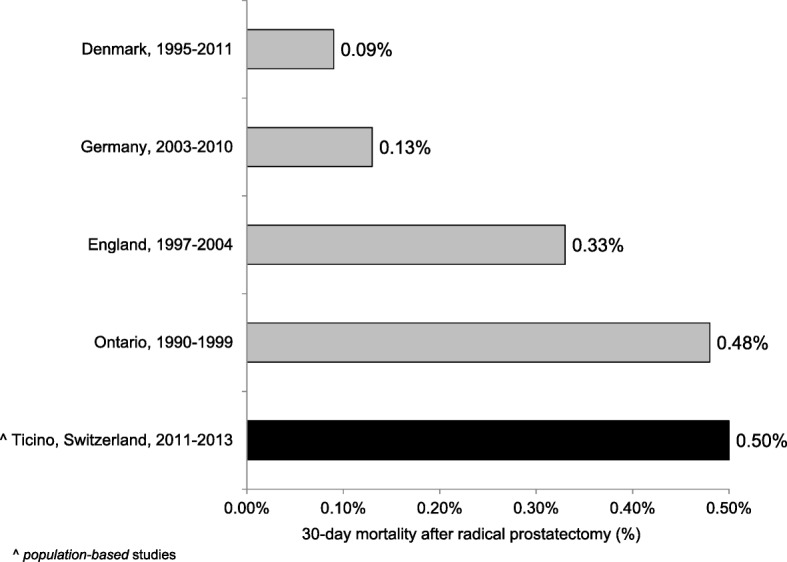
QI13. Proportion of patients with prostate-cancer died within 30-days from radical-prostatectomy: comparison between southern-Switzerland and other countries

- **QI1.** In order to have an accurate staging of the disease and a better treatment planning, the diagnosis should be confirmed histologically [27–33]. In fact, the results of imaging studies, such as computerised tomography scans or magnetic resonance imaging, are not enough to determine the tumour type. In our analysis the transurethral prostatic resection (TUR-P) is not considered as needle-biopsy. Our result is in line with other population-based studies: 84.7 and 78.8% of PC were diagnosed through a transrectal ultrasound guided biopsy, in the regions of South Australia and Victoria and in Denmark respectively [23, 34].
- **QI2.** The ideal number of cores to be taken varies in relation with the volume of the prostatic gland and is still object of many systematic reviews and randomized and non-randomized clinical trials [30, 35–38]. International guidelines recommend taking a minimum of 8 cores but not more than 12, because there is no significant improvement in cancer detection rate [39–41]. In our study 72.5% of patients had 8 or more biopsy cores. Particularly, 65.3% of patients had 8 to 12 biopsy cores, a comparable result to that reported in Spain (64.6%), but lower than in Denmark (78.8%) [34, 42]. Compared to Sweden, where 73% of patients had between 10 and 12 cores taken and 11% between 6 and 9, in southern Switzerland there was a lower proportion of patients with 10–12 biopsy cores (25%) and with 6–9 cores taken (63%) [24]. We believe that a major factor influencing this difference could be the application of different national/international guidelines.
- **QI3–4.** The information reported in the pathology report of needle biopsies and transurethral prostatic resection (TUR-P) are essential for an optimal treatment planning (choice of therapy, risk evaluation linked with the clinical status of the patient, probability to develop distant metastasis) and for the prognosis of PC [43]. According to the guidelines published by the College of American Pathologists, the European Society of Uropathology and the Swiss Society of Pathology, the information about tumour histology, Gleason score and quantification of tissue involved by the tumour have to be specified in the pathology report of needle biopsies and TUR-P [18, 19, 44]. Despite the presence in the literature of many studies assessing the importance of such factors in the therapeutic choices, we could only find a Danish study and two American studies illustrating the proportion of biopsy reports including Gleason score for AJCC stage I-II PC. The reported percentages (97.3, 89.3 and 92.0%) were lower than that of southern Switzerland (100.0%), confirming the elevated quality of work performed from the local institute of pathology [45–47].
- **QI5–6.** As stated for QI3 and QI4, also the standardization of the pathological evaluation of the prostatectomy specimen plays a central role in the PC patients’ care, the adequate treatment decision planning, such as an eventual adjuvant therapy and the prognosis estimation. In the literature we found only two American studies assessing the quality of surgical pathology reporting [48, 49]. As shown in Fig. 1 the results obtained in canton Ticino appeared to be comparable to the U.S.
- **QI7.** There are various options for PC treatment depending on different factors such as stage of the disease, PSA value, Gleason score, as well as patient’s comorbidities and life expectancy. The introduction of PSA screening for the early diagnosis increased the detection of PC that otherwise would have remained silent. To limit the risk of overtreatment, the European guidelines recommend active surveillance as primary treatment for low risk PC [50]. As shown in Fig. 2 there are several studies identifying the proportion of patients with low risk PC undergoing active surveillance, which varies from 16.2% in Germany to 72.0% in Sweden, the only country with a higher proportion than that reported in southern Switzerland [24, 51–55]. Moreover, during more recent years we detected an increase in the use of active surveillance as management option to decrease overtreatment, confirming the agreement to recent guidelines [56, 57].
- **QI8.** Actually, there is no general consensus on the optimal treatment modalities for high risk PC, showing an increased risk of PSA failure and metastatic progression. Specialist’s recommendations and patient’s preferences, especially in relation to side effects of the therapy and their impact on the quality of life, play a relevant role during the choice of the treatment modality. Radical prostatectomy plus pelvic lymphadenectomy as well as radiation therapy plus hormone treatment are the recognized options for high risk PC [57]. Despite the consistent number of missing cases (16.1%), related to the difficulty in retrieving information from the involved clinicians, results from southern Switzerland were comparable with other countries as shown in Fig. 3 [22–24, 53]. There is room for improvement in the harmonization and the collaboration among clinicians, hospitals and cancer registries.
- **QI9.** Guidelines recommend neoadjuvant and concurrent androgen deprivation therapy for patients with high risk PC receiving radical radiotherapy [57]. The value of neoadjuvant therapy is plenty described in randomized trials, such as the TROG trial that showed an improvement in 10-year PC-specific mortality in the group of patients receiving radiotherapy and neoadjuvant hormonal therapy [58]. Despite the presence of many trials evaluating the efficacy and the best duration of neoadjuvant hormonal treatment, there are only few studies, in particular at the population-based level, describing the percentage of high-risk patients undergoing neoadjuvant treatment before radical radiotherapy. Indeed we found out only a population-based Swedish study, which reported a proportion of patients undergoing neoadjuvant treatment equal to 92%, close to 88% observed in southern Switzerland [24].
- **QI10.** The criteria scientifically recognized to evaluate the efficacy of the radical prostatectomy is the margins status of the resected specimen. The percentage of patients with negative margins (R0, i.e. the tumour doesn’t reach the margins) depends on the surgeon’s experience, tumour extension, methods of histologic evaluation and patients’ characteristics such as comorbidities and overweight. By contrast, positive margins (R1) suggest an incomplete resection of the tumour and, therefore, represent an indicator for the execution of adjuvant treatment. Moreover, margins status is an important independent prognostic factor for PC biochemical recurrence and disease-free survival [59–61]. We calculated QI10 separately for PC confined to the prostate (pT2) and PC extended through the prostate capsule (pT3). A recent study conducted at the Ontario Cancer Care recommends to reach a minimum of 75% of patients with negative margins for pT2 disease [62]. Our result is therefore higher than this recommendation, but there is still room for improvement, as shown in Fig. 4 [24, 25, 63–69]. A systematic review of studies published between 2008 and 2011, reported an overall rate of PC with negative surgical margins equal to 91% for pT2 stage (range: 77–96%) and 63% for pT3 stage (range: 50–71%) [70]. In southern Switzerland, the proportion of pT3 PC with R0 was 41.2%, result much lower than that reported in Canada (47.3%), France (65.0%) and Denmark (70.0%, population-based study) [63, 65, 68].
- **QI11.** Radiotherapy is an alternative treatment to radical prostatectomy for localized (M0) PC, warranting the same survival probability and a comparable quality of life compared to surgery. Technical improvements, such as three-dimensional conformal radiotherapy and intensity modulated external-beam techniques, allowed to boost the dose of radiations directly on the tumour volume, reducing the irradiation of the surrounding tissue and the collateral effects. Several randomised and non-randomised trials showed a significant impact of the dose escalation on biochemical recurrence: doses between 74 and 78 Gy, in comparison with 64–70 Gy, increase significantly the progression free survival and extend the time frame before salvage hormonal therapy [71–74]. The European Association of Urology recommends therefore to use a minimum dose greater than 74 Gy, recommendation used to define the QI11 [56]. Despite the plenty of trials analysing the efficacy and related collateral effects of radiotherapy, we could find only two population-based studies to compare our result: both of them reported a lower proportion (66.8% in Sweden and 22.0% in the U.S.) of patients treated with at least 74 Gy than that observed in southern Switzerland (76.3%) [24, 75]
- **QI12.** For metastatic PC the main goals of treatment are to increase survival and prevent/retard the appearance of symptoms due to disease’s progression in order to improve the quality of life. Hormonal therapy is the used method to palliate symptoms and defer progression and disease progression-related complications [56]. The most rapid method to reduce the circulating testosterone level is the bilateral orchiectomy. Pharmacologic castration, obtained through the use of LHRH analogue or antagonist, is reversible (at least partially) and is often psychologically better accepted than orchiectomy. The question whether the beginning of hormonal therapy for asymptomatic patients should be performed immediately after the time of diagnosis or delayed until the biochemical progression is still questioned [56]. In our study we considered the proportion of PC patients undergoing hormonal therapy within three months from the diagnosis, being 73.5%. In the literature we could compare studies considering different time frames between the diagnosis and the beginning of hormonal therapy. The proportion of metastatic PC treated with hormonal therapy varies from 52.0% in Denmark to 88.0% in Sweden and Norway (Fig. 5) [24, 53, 63, 76–78]. We, then, believe that here could be room for improvement in southern Switzerland, since almost a quarter of metastatic PC patients do not receive hormonal therapy within three months from the diagnosis, but even extending the time frame to 6 months or 1 year.
- **QI13.** The postoperative mortality within 30 days after prostatectomy is affected by different factors linked whether with the patient (age, comorbidities, general health status) as with the healthcare system quality and safety [79, 80]. Hospital procedural volume is inversely associated with in-hospital mortality, length of stay and intra- and postoperative complications [81, 82]. The low postoperative mortality observed in southern Switzerland (0.5%) was comparable with the few literature data (see Fig. 6).

## Conclusions

The results of the present study on the QoCC for PC in southern Switzerland give an encouraging and positive picture of the local health care system, although some improvements are still possible. Through this study we assessed the feasibility of data collection and evaluation of QI at the population-based level through cancer registry activity. Standardisation of QI definition as well as further population-based data are needed to set suitable target requirements in order to guarantee a good quality level of the standard of care. The short-term assessment of the diagnostic and therapeutic process through QoCC, could allow taking the necessary measures in the daily clinical practice to ensure an adequate standard of care translating into an immediate benefit for the patients.

## Additional file

Additional file 1: List of QI assessed and selected for prostate cancer, according to the clinical domain. This file describes the full list of selected QI for prostate cancer. (PDF 142 kb)

## Abbreviations

PC: Prostate cancer
QI: Quality indicators
QoCC: Quality of cancer care
TUR-P: Transurethral prostatic resection
