# Supplementary material for: Quality indicators of clinical cancer care for prostate cancer: a population-based study in southern Switzerland
Source: BMC Cancer. 2018 Jul 11;18:733. doi: 10.1186/s12885-018-4604-2 (PMC6042390; doi:10.1186/s12885-018-4604-2)
Supplement: Supplementary file 1 — List of QI assessed and selected for prostate cancer, according to the clinical domain. This file describes the full list of selected QI for prostate cancer. (PDF 142 kb) [file 12885_2018_4604_MOESM1_ESM.pdf]

Additional file 1: list of QI assessed and selected for prostate cancer, according to the clinical domain.

Description	Ref.	Numerator	Denominator
**DIAGNOSIS AND STAGING (n=6)**			
Proportion of patients with prostate cancer and a documented digital rectal examination (DRE).	[1-3]	Number of patients with prostate cancer who had a documented digital rectal examination (DRE).	Number of patients with prostate cancer.
Proportion of patients with prostate cancer and the diagnosis based only on the PSA result.	[2, 4-12]	Number of patients with prostate cancer whose diagnosis was based only on the PSA result.	Number of patients with prostate cancer.
Proportion of patients with prostate cancer and the diagnosis based on prostatic biopsy.	[13-19]	Number of patients with prostate cancer whose diagnosis was based on prostatic biopsy.	Number of patients with prostate cancer.
Proportion of patients with prostate cancer and 8 or more diagnostic prostatic biopsies.	[14, 15, 20]	Number of patients with prostate cancer who underwent 8 or more diagnostic prostatic biopsies.	Number of patients with prostate cancer whose diagnosis was based on prostatic biopsy.
Proportion of patients with prostate cancer and a documented multidisciplinary discussion.	[21-23]	Number of patients with prostate cancer who had a documented multidisciplinary discussion.	Number of patients with prostate cancer.
Proportion of patients with prostate cancer and pre-treatment clinical staging (cTNM) according to the AJCC TNM 7th edition.	[2, 24, 25]	Number of patients with prostate cancer who had a pre-treatment clinical staging (cTNM) according to the AJCC TNM 7th edition.	Number of patients with prostate cancer undergoing curative treatment (surgery or radiotherapy).
**PATHOLOGY (n=4)**			
Proportion of patients with prostate cancer and the pathology report of the biopsy including the following characteristics:<br>- histologic type according to WHO<br>- histologic grade with Gleason score<br>- tumour quantitation (number of positive cores/total number of cores and/or proportion of prostatic tissue involved by tumour).	[26-43]	Number of patients with prostate cancer whose pathology report of the biopsy included the following characteristics:<br>- histologic type according to WHO<br>- histologic grade with Gleason score<br>- tumour quantitation (number of positive cores/total number of cores and/or proportion of prostatic tissue involved by tumour).	Patients with prostate cancer undergoing biopsy.
Proportion of patients with prostate cancer and the pathology report of the TUR-P including the following characteristics:<br>- histologic type according to WHO<br>- histologic grade with Gleason score<br>- tumour quantitation (proportion of prostatic tissue involved by tumour).	[26-43]	Number of patients with prostate cancer whose pathology report of the TUR-P included the following characteristics:<br>- histologic type according to WHO<br>- histologic grade with Gleason score<br>- tumour quantitation (number of positive cores/total number of cores and/or proportion of prostatic tissue involved by tumour).	Patients with prostate cancer undergoing TUR-P.

Proportion of patients with prostate cancer and the pathology report of the prostatectomy with pelvic lymphadenectomy including the number of resected lymph nodes.	[44, 45]	Number of patients with prostate cancer whose pathology report of the prostatectomy with pelvic lymphadenectomy included the number of resected lymph nodes.	Patients with prostate cancer undergoing prostatectomy with pelvic lymphadenectomy.
Proportion of patients with prostate cancer and the pathology report of the prostatectomy (with or without pelvic lymphadenectomy) including the following characteristics:<br>- histologic type according to WHO<br>- histologic grade with Gleason score<br>- extraprostatic extension<br>- seminal vescicle invasion<br>- margins<br>- pathologic staging (pTNM) according to the AJCC TNM 7th edition.	[25-35, 42, 44-57]	Number of patients with prostate cancer whose pathology report of the prostatectomy (with or without pelvic lymphadenectomy) included the following characteristics:<br>- histologic type according to WHO<br>- histologic grade with Gleason score<br>- extraprostatic extension<br>- seminal vescicle invasion<br>- margins<br>- pathologic staging (pTNM) according to the AJCC TNM 7th edition.	Patients with prostate cancer undergoing prostatectomy with or without pelvic lymphadenectomy.
**TREATMENT (n=9)**			
Proportion of patients with localized (N0, M0) low risk (cT1-2a and Gleason≤6 and PSA≤10 ng/ml) prostate cancer, stratified according to the 1st treatment received (no treatment, active surveillance, radical prostatectomy ± pelvic lymphadenectomy, HT, RT, ChT).	[58-61]	Number of patients with localized (N0, M0) low risk (cT1-2a and Gleason≤6 and PSA≤10 ng/ml) prostate cancer who underwent the following 1st treatment:<br>- no treatment<br>- active surveillance<br>- radical prostatectomy ± pelvic lymphadenectomy<br>- HT<br>- RT<br>- ChT.	Number of patients with localized (N0, M0) low risk (cT1-2a and Gleason≤6 and PSA≤10 ng/ml) prostate cancer.
Proportion of patients with localized (N0, M0) intermediate risk (cT2b-2c or Gleason=7 or PSA 10-20 ng/ml) prostate cancer, stratified according to the 1st treatment received (no treatment, active surveillance, radical prostatectomy ± pelvic lymphadenectomy, HT, RT, ChT).	[59-62]	Number of patients with localized (N0, M0) intermediate risk (cT2b-2c or Gleason=7 or PSA 10-20 ng/ml) prostate cancer who underwent the following 1st treatment:<br>- no treatment<br>- active surveillance<br>- radical prostatectomy ± pelvic lymphadenectomy<br>- HT<br>- RT<br>- ChT.	Number of patients with localized (N0, M0) intermediate risk (cT2b-2c or Gleason=7 or PSA 10-20 ng/ml) prostate cancer.
Proportion of patients with localized (N0, M0) high risk (cT3-4 or Gleason≥8 or PSA≥20 ng/ml) prostate cancer, stratified according to the 1st treatment received (no treatment, active surveillance, radical prostatectomy ± pelvic lymphadenectomy, HT, RT, ChT).	[59, 62-70]	Number of patients with localized (N0, M0) high risk (cT3-4 or Gleason≥8 or PSA≥20 ng/ml) prostate cancer who underwent the following 1st treatment:<br>- no treatment<br>- active surveillance<br>- radical prostatectomy ± pelvic lymphadenectomy<br>- HT<br>- RT<br>- ChT.	Number of patients with localized (N0, M0) high risk (cT3-4 or Gleason≥8 or PSA≥20 ng/ml) prostate cancer.
Proportion of patients with localized (N0, M0) low risk (cT1-2a and Gleason≤6 and PSA≤10 ng/ml) prostate cancer undergoing active surveillance.	[58-61, 71-75]	Number of patients with localized (N0, M0) low risk (cT1-2a and Gleason≤6 and PSA≤10 ng/ml) prostate cancer who underwent an active surveillance.	Number of patients with localized (N0, M0) low risk (cT1-2a and Gleason≤6 and PSA≤10 ng/ml) prostate cancer.

Proportion of patients with localized (N0, M0) high risk (cT3-4 or Gleason≥8 or PSA≥20 ng/ml) prostate cancer undergoing radical treatment (radical prostatectomy ± pelvic lymphadenectomy, RT or brachytherapy).	[59-62]	Number of patients with localized (N0, M0) high risk (cT3-4 or Gleason≥8 or PSA≥20 ng/ml) prostate cancer who underwent a radical treatment (radical prostatectomy ± pelvic lymphadenectomy, RT or brachytherapy).	Number of patients with localized (N0, M0) high risk (cT3-4 or Gleason≥8 or PSA≥20 ng/ml) prostate cancer.
Proportion of patients with localized (N0, M0) high risk (cT3-4 or Gleason≥8 or PSA≥20 ng/ml) prostate cancer undergoing radical RT with neo-adjuvant HT.	[76-79]	Number of patients with localized (N0, M0) high risk (cT3-4 or Gleason≥8 or PSA≥20 ng/ml) prostate cancer who underwent a neo-adjuvant HT before radical RT.	Number of patients with localized (N0, M0) high risk (cT3-4 or Gleason≥8 or PSA≥20 ng/ml) prostate cancer undergoing radical RT.
Proportion of patients with pT2 or pT3 prostate cancer undergoing prostatectomy ± pelvic lymphadenectomy with uninvolved margins.	[47-52, 80, 81]	Number of patients with pT2 or pT3 prostate cancer who underwent prostatectomy ± pelvic lymphadenectomy with uninvolved margins.	Number of patients with pT2 or pT3 prostate cancer undergoing prostatectomy ± pelvic lymphadenectomy.
Proportion of patients with non-metastatic (M0) prostate cancer undergoing external beam RT with dose escalation to at least 74 Gy.	[82-85]	Number of patients with non-metastatic (M0) prostate cancer who underwent an external beam RT with dose escalation to at least 74 Gy.	Number of patients with non-metastatic (M0) prostate cancer undergoing RT.
Proportion of patients with metastatic (M1) prostate cancer undergoing immediate (within 3 months form the diagnosis) HT or bilateral orchiectomy.	[86, 87]	Number of patients with metastatic (M1) prostate cancer who underwent an immediate (within 3 months from the diagnosis) HT or a bilateral orchiectomy.	Patients with metastatic (M1) prostate cancer.
**FOLLOW-UP AND OUTCOME (n=4)**			
Analysis of disease-free survival at 1, 3 and 5 years from curative treatment.	[2, 88]	Number of patients with prostate cancer who were disease-free at 1, 3 and 5 years from curative treatment.	Number of patients with prostate cancer undergoing radical treatment (surgery or radiotherapy).
Proportion of patients with prostate cancer and PSA relapse (<0.2 ng/ml) within 1 year after radical prostatectomy ± pelvic lymphadenectomy.	[2]	Number of patients with prostate cancer who had a PSA relapse (<0.2 ng/ml) within 1 year after radical prostatectomy ± pelvic lymphadenectomy.	Number of patients with prostate cancer undergoing radical prostatectomy ± pelvic lymphadenectomy.
Analysis of overall survival at 1, 3 and 5 years from diagnosis.		Number of patients with prostate cancer who survived at 1, 3 and 5 years from diagnosis.	Number of patients with prostate cancer.
Proportion of patients with non-metastatic (M0) prostate cancer dead within 30 days after prostatectomy ± pelvic lymphadenectomy (post-operative mortality).	[2, 89]	Number of patients with non-metastatic (M0) prostate cancer who died within 30 days after prostatectomy ± pelvic lymphadenectomy.	Number of patients with non-metastatic (M0) prostate cancer undergoing prostatectomy ± pelvic lymphadenectomy.

Abbreviation:

PSA= Prostate Specific Antigen; **TUR-P**= transurethral prostatic resection; **AJCC**= America Joint Committee on Cancer; **WHO**= World Health Organization; **HT**= Hormonal Therapy; **RT**=Radiotherapy; **ChT**= Chemotherapy.

References

1. Carvalhal GF, Smith DS, Mager DE, Ramos C, Catalona WJ: Digital rectal examination for detecting prostate cancer at prostate specific antigen levels of 4 ng./ml. or less. J Urol 1999, 161:835-839.
2. Spencer BA, Steinberg M, Malin J, Adams J, Litwin MS: Quality-of-care indicators for early-stage prostate cancer. J Clin Oncol 2003, 21:1928-1936.
3. Horwick A, Parker C, Bangma CH, Kataya V: Prostate Cancer: ESMO Clinical Practice Guidelines for diagnosis, treatment and follow-up. Ann Oncol 2010, 21:v129-v133.
4. Catalona WJ, Richie JP, Ahmann FR, Hudson MA, Scardino PT, Flanigan RC, deKernion JB, Ratliff TL, Kavoussi LR, Dalkin BL, et al.: Comparison of digital rectal examination and serum prostate specific antigen in the early detection of prostate cancer: results of a multicenter clinical trial of 6,630 men. J Urol 1994, 151:1283-1290.
5. Schroder FH, Hugosson J, Roobol MJ, Tammela TL, Ciatto S, Nelen V, Kwiatkowski M, Lujan M, Lilja H, Zappa M, et al: Screening and prostate-cancer mortality in a randomized European study. N Engl J Med 2009, 360:1320-1328.
6. Andriole GL, Crawford ED, Grubb RL, 3rd, Buys SS, Chia D, Church TR, Fouad MN, Gelmann EP, Kvale PA, Reding DJ, et al: Mortality results from a randomized prostate-cancer screening trial. N Engl J Med 2009, 360:1310-1319.
7. Thompson IM, Ankerst DP, Chi C, Goodman PJ, Tangen CM, Lucia MS, Feng Z, Parnes HL, Coltman CA, Jr.: Assessing prostate cancer risk: results from the Prostate Cancer Prevention Trial. J Natl Cancer Inst 2006, 98:529-534.
8. Eastham JA, Riedel E, Scardino PT, Shike M, Fleisher M, Schatzkin A, Lanza E, Latkany L, Begg CB: Variation of serum prostate-specific antigen levels: an evaluation of year-to-year fluctuations. JAMA 2003, 289:2695-2700.
9. Thompson IM, Pauler DK, Goodman PJ, Tangen CM, Lucia MS, Parnes HL, Minasian LM, Ford LG, Lippman SM, Crawford ED, et al: Prevalence of prostate cancer among men with a prostate-specific antigen level < or =4.0 ng per milliliter. N Engl J Med 2004, 350:2239-2246.
10. Stamey TA, Yang N, Hay AR, McNeal JE, Freiha FS, Redwine E: Prostate-specific antigen as a serum marker for adenocarcinoma of the prostate. N Engl J Med 1987, 317:909-916.
11. Carter HB, Pearson JD, Metter EJ, Brant LJ, Chan DW, Andres R, Fozard JL, Walsh PC: Longitudinal evaluation of prostate-specific antigen levels in men with and without prostate disease. JAMA 1992, 267:2215-2220.
12. Schmid HP, McNeal JE, Stamey TA: Observations on the doubling time of prostate cancer. The use of serial prostate-specific antigen in patients with untreated disease as a measure of increasing cancer volume. Cancer 1993, 71:2031-2040.
13. Babaian RJ, Toi A, Kamoi K, Troncoso P, Sweet J, Evans R, Johnston D, Chen M: A comparative analysis of sextant and an extended 11-core multisite directed biopsy strategy. J Urol 2000, 163:152-157.
14. Hara R, Jo Y, Fujii T, Kondo N, Yokoyoma T, Miyaji Y, Nagai A: Optimal approach for prostate cancer detection as initial biopsy: prospective randomized study comparing transperineal versus transrectal systematic 12-core biopsy. Urology 2008, 71:191-195.
15. Takenaka A, Hara R, Ishimura T, Fujii T, Jo Y, Nagai A, Fujisawa M: A prospective randomized comparison of diagnostic efficacy between transperineal and transrectal 12-core prostate biopsy. Prostate Cancer Prostatic Dis 2008, 11:134-138.
16. Eichler K, Hempel S, Wilby J, Myers L, Bachmann LM, Kleijnen J: Diagnostic value of systematic biopsy methods in the investigation of prostate cancer: a systematic review. J Urol 2006, 175:1605-1612.
17. Donovan J, Hamdy F, Neal D, Peters T, Oliver S, Brindle L, Jewell D, Powell P, Gillatt D, Dedman D, et al: Prostate Testing for Cancer and Treatment (ProtecT) feasibility study. Health Technol Assess 2003, 7:1-88.
18. Aron M, Rajeev TP, Gupta NP: Antibiotic prophylaxis for transrectal needle biopsy of the prostate: a randomized controlled study. BJU Int 2000, 85:682-685.
19. Presti JC, Jr., O'Dowd GJ, Miller MC, Mattu R, Veltri RW: Extended peripheral zone biopsy schemes increase cancer detection rates and minimize variance in prostate specific antigen and age related cancer rates: results of a community multi-practice study. J Urol 2003, 169:125-129.

20. Suzuki M, Kawakami S, Asano T, Masuda H, Saito K, Koga F, Fujii Y, Kihara K: Safety of transperineal 14-core systematic prostate biopsy in diabetic men. Int J Urol 2009, 16:930-935.
21. Kurpad R, Kim W, Kim Rathmell W, Godley P, Whang Y, Fielding J, Smith L, Pettiford A, Schultz H, Nielsen M, et al: A multidisciplinary approach to the management of urologic malignancies: Does it influence diagnostic and treatment decisions? Urol Oncol 2009.
22. Bauman G, Winquist E, Chin J: A pilot study of regional participation in a videoconferenced multidisciplinary genitourinary tumor board. Can J Urol 2005, 12:2532-2536.
23. Valdagni R, Albers P, Bangma C, Drudge-Coates L, Magnani T, Moynihan C, Parker C, Redmond K, Sternberg CN, Denis L, Costa A: The requirements of a specialist Prostate Cancer Unit: a discussion paper from the European School of Oncology. Eur J Cancer 2011, 47:1-7.
24. Heidenreich A, Pfister D, Thuer D, Brehmer B: Percentage of positive biopsies predicts lymph node involvement in men with low-risk prostate cancer undergoing radical prostatectomy and extended pelvic lymphadenectomy. BJU Int 2011, 107:220-225.
25. Edge SB, Byrd DR, Carducci MA, Compton CC (Eds.): AJCC Cancer Staging Manual, 7th edition: Springer-Verlag, New York, NY; 2010.
26. Srigley JR, Humphrey PA, Amin MB, Chang SS, Egevad LL, Epstein JI, Grignon DJ, McKieman JM, Montironi R, Renshaw AA, et al: Protocol for the examination of specimens from patients with carcinoma of the prostate gland. (Pathologists CoA ed.; 2009.
27. Young RH, Srigley JR, Amin MB, Ulbright TM, Cubilla A: Tumors of the prostate gland, seminal vesicle, male urethra and penis. Washington D.C.: Armed Forces Institute of Pathology; 2000.
28. Gleason DF, Mellinger GT: Prediction of prognosis for prostatic adenocarcinoma by combined histological grading and clinical staging. J Urol 1974, 111:58-64.
29. Amin MB, Grignon DJ, Humphreys EB, Srigley JR: Gleason grading of prostate cancer. A contemporary approach. Philadelphia, PA: Lippincott Williams & Wilkins; 2004.
30. Stephenson AJ, Scardino PT, Eastham JA, Bianco FJ, Jr., Dotan ZA, Fearn PA, Kattan MW: Preoperative nomogram predicting the 10-year probability of prostate cancer recurrence after radical prostatectomy. J Natl Cancer Inst 2006, 98:715-717.
31. Stephenson AJ, Scardino PT, Eastham JA, Bianco FJ, Jr., Dotan ZA, DiBlasio CJ, Reuther A, Klein EA, Kattan MW: Postoperative nomogram predicting the 10-year probability of prostate cancer recurrence after radical prostatectomy. J Clin Oncol 2005, 23:7005-7012.
32. Makarov DV, Trock BJ, Humphreys EB, Mangold LA, Walsh PC, Epstein JI, Partin AW: Updated nomogram to predict pathologic stage of prostate cancer given prostate-specific antigen level, clinical stage, and biopsy Gleason score (Partin tables) based on cases from 2000 to 2005. Urology 2007, 69:1095-1101.
33. Epstein JI, Allsbrook WC, Jr., Amin MB, Egevad LL: The 2005 International Society of Urological Pathology (ISUP) Consensus Conference on Gleason Grading of Prostatic Carcinoma. Am J Surg Pathol 2005, 29:1228-1242.
34. Patel AA, Chen MH, Renshaw AA, D'Amico AV: PSA failure following definitive treatment of prostate cancer having biopsy Gleason score 7 with tertiary grade 5. JAMA 2007, 298:1533-1538.
35. Amin M, Boccon-Gibod L, Egevad LL: Prognostic and predictive factors and reporting of prostate carcinoma in prostate needle biopsy specimens. (2005 WHO-sponseored International Consultation Consensus). Scand J Urol Nephrol 2004, 2004:20-33.
36. Cupp MR, Bostwick DG, Myers RP, Oesterling JE: The volume of prostate cancer in the biopsy specimen cannot reliably predict the quantity of cancer in the radical prostatectomy specimen on an individual basis. J Urol 1995, 153:1543-1548.
37. Ravery V, Boccon-Gibod LA, Dauge-Geffroy MC, Billebaud T, Delmas V, Meulemans A, Toublanc M, Boccon-Gibod L: Systematic biopsies accurately predict extracapsular extension of prostate cancer and persistent/recurrent detectable PSA after radical prostatectomy. Urology 1994, 44:371-376.
38. Ravery V, Schmid HP, Toublanc M, Boccon-Gibod L: Is the percentage of cancer in biopsy cores predictive of extracapsular disease in T1-T2 prostate carcinoma? Cancer 1996, 78:1079-1084.
39. Freedland SJ, Csathy GS, Dorey F, Aronson WJ: Percent prostate needle biopsy tissue with cancer is more predictive of biochemical failure or adverse pathology after radical prostatectomy than prostate specific antigen or Gleason score. J Urol 2002, 167:516-520.
40. Bismar TA, Lewis JS, Jr., Vollmer RT, Humphrey PA: Multiple measures of carcinoma extent versus perineural invasion in prostate needle biopsy tissue in prediction of pathologic stage in a screening population. Am J Surg Pathol 2003, 27:432-440.

41. Stamey TA, Freiha FS, McNeal JE, Redwine EA, Whittemore AS, Schmid HP: Localized prostate cancer. Relationship of tumor volume to clinical significance for treatment of prostate cancer. Cancer 1993, 71:933-938.
42. Renshaw AA, Richie JP, Loughlin KR, Jiroutek M, Chung A, D'Amico AV: Maximum diameter of prostatic carcinoma is a simple, inexpensive, and independent predictor of prostate-specific antigen failure in radical prostatectomy specimens. Validation in a cohort of 434 patients. Am J Clin Pathol 1999, 111:641-644.
43. Humphrey PA, Vollmer RT: Percentage carcinoma as a measure of prostatic tumor size in radical prostatectomy tissues. Mod Pathol 1997, 10:326-333.
44. Srigley JR: Key issues in handling and reporting radical prostatectomy specimens. Arch Pathol Lab Med 2006, 130:303-317.
45. Sehdev AE, Pan CC, Epstein JI: Comparative analysis of sampling methods for grossing radical prostatectomy specimens performed for nonpalpable (stage T1c) prostatic adenocarcinoma. Hum Pathol 2001, 32:494-499.
46. Wheeler TM, Dillioglugil O, Kattan MW, Arakawa A, Soh S, Suyama K, Ohori M, Scardino PT: Clinical and pathological significance of the level and extent of capsular invasion in clinical stage T1-2 prostate cancer. Hum Pathol 1998, 29:856-862.
47. Ohori M, Kattan M, Scardino PT, Wheeler TM: Radical prostatectomy for carcinoma of the prostate. Mod Pathol 2004, 17:349-359.
48. Epstein JI, Amin MB, Boccon-Gibod L: Prognostic factors and reporting of prostate carcinoma in radical prostectomy and pelvic lymphadenectomy specimens. Scand J Urol Nephrol 2005, 216:34-63.
49. Epstein JI, Sauvageot J: Do close but negative margins in radical prostatectomy specimens increase the risk of postoperative progression? J Urol 1997, 157:241-243.
50. Graefen M, Hammerer P, Michl U, Noldus J, Haese A, Henke RP, Huland E, Huland H: Incidence of positive surgical margins after biopsy-selected nerve-sparing radical prostatectomy. Urology 1998, 51:437-442.
51. Ohori M, Wheeler TM, Kattan MW, Goto Y, Scardino PT: Prognostic significance of positive surgical margins in radical prostatectomy specimens. J Urol 1995, 154:1818-1824.
52. Epstein JI, Partin AW, Sauvageot J, Walsh PC: Prediction of progression following radical prostatectomy. A multivariate analysis of 721 men with long-term follow-up. Am J Surg Pathol 1996, 20:286-292.
53. Greene FL, Compton CC, A.G. f, Shah J, D.P. W (Eds.): AJCC Cancer Staging Atlas. New York, NY: Springer; 2006.
54. Epstein JI, Herawi M: Prostate needle biopsies containing prostatic intraepithelial neoplasia or atypical foci suspicious for carcinoma: implications for patient care. J Urol 2006, 175:820-834.
55. Netto GJ, Epstein JI: Widespread high-grade prostatic intraepithelial neoplasia on prostatic needle biopsy: a significant likelihood of subsequently diagnosed adenocarcinoma. Am J Surg Pathol 2006, 30:1184-1188.
56. Gokden N, Roehl KA, Catalona WJ, Humphrey PA: High-grade prostatic intraepithelial neoplasia in needle biopsy as risk factor for detection of adenocarcinoma: current level of risk in screening population. Urology 2005, 65:538-542.
57. Merrimen JL, Jones G, Walker D, Leung CS, Kapusta LR, Srigley JR: Multifocal high grade prostatic intrahepitelial neoplasia is a significant risk factor for prostatic adenocarcinoma. J Urol 2009, 183:485-490.
58. Johansson JE, Andren O, Andersson SO, Dickman PW, Holmberg L, Magnuson A, Adami HO: Natural history of early, localized prostate cancer. JAMA 2004, 291:2713-2719.
59. D'Amico AV, Whittington R, Malkowicz SB, Schultz D, Blank K, Broderick GA, Tomaszewski JE, Renshaw AA, Kaplan I, Beard CJ, Wein A: Biochemical outcome after radical prostatectomy, external beam radiation therapy, or interstitial radiation therapy for clinically localized prostate cancer. JAMA 1998, 280:969-974.
60. Hayes JH, Ollendorf DA, Pearson SD, Barry MJ, Kantoff PW, Stewart ST, Bhatnagar V, Sweeney CJ, Stahl JE, McMahon PM: Active surveillance compared with initial treatment for men with low-risk prostate cancer: a decision analysis. JAMA 2010, 304:2373-2380.
61. Weckermann D, Goppelt M, Dorn R, Wawroschek F, Harzmann R: Incidence of positive pelvic lymph nodes in patients with prostate cancer, a prostate-specific antigen (PSA) level of < or =10 ng/mL and biopsy Gleason score of < or =6, and their influence on PSA progression-free survival after radical prostatectomy. BJU Int 2006, 97:1173-1178.

62. Martinez AA, Gonzalez J, Ye H, Ghilezan M, Shetty S, Kernen K, Gustafson G, Krauss D, Vicini F, Kestin L: Dose escalation improves cancer-related events at 10 years for intermediate- and high-risk prostate cancer patients treated with hypofractionated high-dose-rate boost and external beam radiotherapy. Int J Radiat Oncol Biol Phys 2011, 79:363-370.
63. Lau WK, Bergstralh EJ, Blute ML, Slezak JM, Zincke H: Radical prostatectomy for pathological Gleason 8 or greater prostate cancer: influence of concomitant pathological variables. J Urol 2002, 167:117-122.
64. Thompson IM, Tangen CM, Paradelo J, Lucia MS, Miller G, Troyer D, Messing E, Forman J, Chin J, Swanson G, et al: Adjuvant radiotherapy for pathological T3N0M0 prostate cancer significantly reduces risk of metastases and improves survival: long-term followup of a randomized clinical trial. J Urol 2009, 181:956-962.
65. Boorjian SA, Karnes RJ, Viterbo R, Rangel LJ, Bergstralh EJ, Horwitz EM, Blute ML, Buyyounouski MK: Long-term survival after radical prostatectomy versus external-beam radiotherapy for patients with high-risk prostate cancer. Cancer 2011, 117:2883-2891.
66. Thoms J, Goda JS, Zlotta AR, Fleshner NE, van der Kwast TH, Supiot S, Warde P, Bristow RG: Neoadjuvant radiotherapy for locally advanced and high-risk prostate cancer. Nat Rev Clin Oncol 2011, 8:107-113.
67. Bolla M, Van Tienhoven G, Warde P, Dubois JB, Mirimanoff RO, Storme G, Bernier J, Kuten A, Sternberg C, Billiet I, et al: External irradiation with or without long-term androgen suppression for prostate cancer with high metastatic risk: 10-year results of an EORTC randomised study. Lancet Oncol 2010, 11:1066-1073.
68. Rosenthal MA, Davidson P, Rolland F, Campone M, Xue L, Han TH, Mehta A, Berd Y, He W, Lombardi A: Evaluation of the safety, pharmacokinetics and treatment effects of an alpha(nu)beta(3) integrin inhibitor on bone turnover and disease activity in men with hormone-refractory prostate cancer and bone metastases. Asia Pac J Clin Oncol 2010, 6:42-48.
69. Zapatero A, Garcia-Vicente F, Martin de Vidales C, Cruz Conde A, Ibanez Y, Fernandez I, Rabadan M: Long-Term Results After High-Dose Radiotherapy and Adjuvant Hormones in Prostate Cancer: How Curable Is High-Risk Disease? Int J Radiat Oncol Biol Phys 2010.
70. Parikh R, Sher DJ: Primary radiotherapy versus radical prostatectomy for high-risk prostate cancer: A decision analysis. Cancer 2011.
71. Coen JJ, Feldman AS, Smith MR, Zietman AL: Watchful waiting for localized prostate cancer in the PSA era: what have been the triggers for intervention? BJU Int 2010.
72. Albertsen PC, Hanley JA, Fine J: 20-year outcomes following conservative management of clinically localized prostate cancer. JAMA 2005, 293:2095-2101.
73. Albertsen PC, Hanley JA, Gleason DF, Barry MJ: Competing risk analysis of men aged 55 to 74 years at diagnosis managed conservatively for clinically localized prostate cancer. JAMA 1998, 280:975-980.
74. Chodak GW, Thisted RA, Gerber GS, Johansson JE, Adolfsson J, Jones GW, Chisholm GD, Moskovitz B, Livne PM, Warner J: Results of conservative management of clinically localized prostate cancer. N Engl J Med 1994, 330:242-248.
75. Bill-Axelson A, Holmberg L, Filen F, Ruutu M, Garmo H, Busch C, Nordling S, Haggman M, Andersson SO, Bratell S, et al: Radical prostatectomy versus watchful waiting in localized prostate cancer: the Scandinavian prostate cancer group-4 randomized trial. J Natl Cancer Inst 2008, 100:1144-1154.
76. D'Amico AV, Chen MH, Renshaw AA, Loffredo M, Kantoff PW: Androgen suppression and radiation vs radiation alone for prostate cancer: a randomized trial. JAMA 2008, 299:289-295.
77. Deutsch I, Zelefsky MJ, Zhang Z, Mo Q, Zaider M, Cohen G, Cahlon O, Yamada Y: Comparison of PSA relapse-free survival in patients treated with ultra-high-dose IMRT versus combination HDR brachytherapy and IMRT. Brachytherapy 2010, 9:313-318.
78. Bolla M, Fourneret P, Beneyton V, Tessier A, Jover F, Verry C: [Combination of external irradiation and androgen suppression for prostate cancer: facts and questions]. Cancer Radiother 2010, 14:510-514.
79. Horwitz EM, Bae K, Hanks GE, Porter A, Grignon DJ, Brereton HD, Venkatesan V, Lawton CA, Rosenthal SA, Sandler HM, Shipley WU: Ten-year follow-up of radiation therapy oncology group protocol 92-02: a phase III trial of the duration of elective androgen deprivation in locally advanced prostate cancer. J Clin Oncol 2008, 26:2497-2504.
80. Song C, Kang T, Yoo S, Jeong IG, Ro JY, Hong JH, Kim CS, Ahn H: Tumor volume, surgical margin, and the risk of biochemical recurrence in men with organ-confined prostate cancer. Urol Oncol 2011.

81. Kench JG, Clouston DR, Delprado W, Eade T, Ellis D, Horvath LG, Samaratunga H, Stahl J, Stapleton AM, Egevad L, et al: Prognostic factors in prostate cancer. Key elements in structured histopathology reporting of radical prostatectomy specimens. Pathology 2011.
82. Dearnaley DP, Hall E, Lawrence D, Huddart RA, Eeles R, Nutting CM, Gadd J, Warrington A, Bidmead M, Horwich A: Phase III pilot study of dose escalation using conformal radiotherapy in prostate cancer: PSA control and side effects. Br J Cancer 2005, 92:488-498.
83. Zietman AL, Bae K, Slater JD, Shipley WU, Efstathiou JA, Coen JJ, Bush DA, Lunt M, Spiegel DY, Skowronski R, et al: Randomized trial comparing conventional-dose with high-dose conformal radiation therapy in early-stage adenocarcinoma of the prostate: long-term results from proton radiation oncology group/american college of radiology 95-09. J Clin Oncol 2010, 28:1106-1111.
84. Eade TN, Hanlon AL, Horwitz EM, Buyyounouski MK, Hanks GE, Pollack A: What dose of external-beam radiation is high enough for prostate cancer? Int J Radiat Oncol Biol Phys 2007, 68:682-689.
85. Kuban DA, Tucker SL, Dong L, Starkschall G, Huang EH, Cheung MR, Lee AK, Pollack A: Long-term results of the M. D. Anderson randomized dose-escalation trial for prostate cancer. Int J Radiat Oncol Biol Phys 2008, 70:67-74.
86. Heidenreich A, Bastian PJ, Bellmunt J, Bolla M, Joniau S, van der Kwast T, Mason M, Matveev V, Wiegel T, Zattoni F, Mottet N: EAU guidelines on prostate cancer. Part II: Treatment of advanced, relapsing, and castration-resistant prostate cancer. Eur Urol 2014, 65:467-479.
87. Immediate versus deferred treatment for advanced prostatic cancer: initial results of the Medical Research Council Trial. The Medical Research Council Prostate Cancer Working Party Investigators Group. Br J Urol 1997, 79:235-246.
88. Johansson JE, Holmberg L, Johansson S, Bergstrom R, Adami HO: Fifteen-year survival in prostate cancer. A prospective, population-based study in Sweden. JAMA 1997, 277:467-471.
89. Miller DC, Litwin MS, Sanda MG, Montie JE, Dunn RL, Resh J, Sandler H, Wei JT: Use of quality indicators to evaluate the care of patients with localized prostate carcinoma. Cancer 2003, 97:1428-1435.
